# Dissecting the role of the Tir:Nck and Tir:IRTKS/IRSp53 signalling pathways *in vivo*

**DOI:** 10.1111/j.1365-2958.2009.06938.x

**Published:** 2009-11-25

**Authors:** Valérie F Crepin, Francis Girard, Stephanie Schüller, Alan D Phillips, Aurelie Mousnier, Gad Frankel

**Affiliations:** 1 Centre for Molecular Microbiology and Infection, Division of Cell and Molecular Biology, Imperial College LondonLondon, UK; 2 Centre for Paediatric Gastroenterology, Royal Free HospitalLondon, UK

## Abstract

Attaching and effacing (A/E) lesions and actin polymerization, the hallmark of enteropathogenic *Escherichia coli* (EPEC), enterohemorrhagic *E. coli* (EHEC) and *Citrobacter rodentium* (CR) infections, are dependent on the effector Tir. Phosphorylation of Tir_EPEC/CR_ Y474/1 leads to recruitment of Nck and neural Wiskott–Aldrich syndrome protein (N-WASP) and strong actin polymerization in cultured cells. Tir_EPEC/CR_ also contains an Asn-Pro-Tyr (NPY_454/1_) motif, which triggers weak actin polymerization. In EHEC the NPY_458_ actin polymerization pathway is amplified by TccP/EspF_U_, which is recruited to Tir via IRSp53 and/or insulin receptor tyrosine kinase substrate (IRTKS). Here we used *C. rodentium* to investigate the different Tir signalling pathways *in vivo*. Following infection with wild-type *C. rodentium* IRTKS, but not IRSp53, was recruited to the bacterial attachment sites. Similar results were seen after infection of human ileal explants with EHEC. Mutating Y471 or Y451 in Tir_CR_ abolished recruitment of Nck and IRTKS respectively, but did not affect recruitment of N-WASP or A/E lesion formation. This suggests that despite their crucial role in actin polymerization in cultured cells the Tir:Nck and Tir:IRTKS pathways are not essential for N-WASP recruitment or A/E lesion formation *in vivo*. Importantly, wild-type *C. rodentium* out-competed the *tir* tyrosine mutants during mixed infections. These results uncouple the Tir:Nck and Tir:IRTKS pathways from A/E lesion formation *in vivo* but assign them an important *in vivo* role.

## Introduction

Enteropathogenic *Escherichia coli* (EPEC) and enterohemorrhagic *E. coli* (EHEC), particularly serotype O157:H7, are important human pathogens (Nataro and Kaper, 1998). EPEC is an important cause of infantile diarrhoea in developing countries, while EHEC is a major food-borne pathogen in developed countries that can cause bloody diarrhoea, haemorrhagic colitis and haemolytic uremic syndrome, haemolytic uremic syndrome being the leading cause of paediatric kidney failure in the USA and UK (Nataro and Kaper, 1998). *Citrobacter rodentium* is a mouse-specific pathogen, the ethiological agent of transmissible colonic hyperplasia, and a model EPEC and EHEC microorganism (Mundy *et al.*, 2005).

While colonizing the gut mucosa EPEC, EHEC and *C. rodentium* induce attaching and effacing (A/E) lesions, which are characterized by extensive remodelling of the gut epithelium leading to elongation and effacement of the brush border microvilli, intimate bacterial attachment to the enterocyte apical plasma membrane, accumulation of polymerized actin and formation of elevated pedestal-like structures (Knutton *et al.*, 1987). Adhesion of EPEC, EHEC (reviewed in Frankel *et al.*, 1998; Frankel and Phillips, 2008) and *C. rodentium* (Girard *et al.*, 2009a) to cultured cells also triggers actin polymerization under attached bacteria. For this reason dissecting the actin signalling pathways *in vitro* was at the heart of EPEC and EHEC research for over two decades.

The ability to induce A/E lesions and actin polymerization is encoded within the locus of enterocyte effacement (McDaniel *et al.*, 1995). The locus of enterocyte effacement encodes a type III secretion system (Jarvis *et al.*, 1995), the outer membrane adhesin intimin (Jerse *et al.*, 1990), regulators, chaperones and translocator and effector proteins (reviewed in Garmendia *et al.*, 2005), including Tir (Kenny *et al.*, 1997). Following translocation, Tir is integrated into the epithelial cell plasma membrane in a hairpin loop topology (Hartland *et al.*, 1999), exposing an extracellular central domain that functions as an intimin receptor. Binding of intimin induces clustering of Tir, assembly of signalling complexes and actin polymerization (reviewed in Caron *et al.*, 2006).

Using cultured epithelial cells and the prototype EPEC strain E2348/69 have shown that actin polymerization is predominantly dependent on phosphorylation of the C-terminal Tir tyrosine residue 474 (Y474) (Kenny, 1999). It was recently reported that a short polyproline sequence at the N-terminus of Tir_EPEC_ is needed for recruitment of redundant tyrosine kinases that phosphorylate Y474; substitution of the polyprolines with alanines prevented actin polymerization (Bommarius *et al.*, 2007). Phosphorylation of Tir residue Y474 provides a binding site for the mammalian adaptor protein Nck (Gruenheid *et al.*, 2001; Campellone *et al.*, 2002; 2004a). Recruitment of Nck leads to activation of the neural Wiskott–Aldrich syndrome protein (N-WASP), recruitment of the Arp2/3 complex and actin polymerization (reviewed in Caron *et al.*, 2006). In addition, Tir_EPEC_ can promote weak actin polymerization, in an Nck-independent manner, involving the C-terminal Tir tyrosine residue Y454 (Campellone and Leong, 2005), which is present in the context of a conserved Asn-Pro-Tyr (NPY) motif (Brady *et al.*, 2007).

In contrast to Tir_EPEC_, Tir_EHEC_ contains the NPY motif but lacks the Nck binding site (Brady *et al.*, 2007). Nevertheless, EHEC O157:H7 triggers strong actin polymerization as it additionally translocates TccP/EspF_U_, which is recruited to Tir though indirectly (Campellone *et al.*, 2004b; Garmendia *et al.*, 2004). Recently, Vingadassalom *et al.* (2009) have shown that the Tir NPY motif recruits the adaptor protein insulin receptor tyrosine kinase substrate (IRTKS), while Weiss *et al.* (2009) have shown that rather than IRTKS Tir recruits the insulin receptor substrate protein of 53 kDa (IRSp53). IRTKS and IRSp53 were shown to link Tir and TccP/EspF_U_, which in turn activates N-WASP (Garmendia *et al.*, 2006; Campellone *et al.*, 2008; Cheng *et al.*, 2008; Sallee *et al.*, 2008), leading to actin polymerization.

Importantly, EPEC strains belonging to lineage 2 and non-O157 EHEC strains can simultaneously trigger actin polymerization by the Tir:Nck and Tir:TccP/TccP2 pathways (Whale *et al.*, 2006; 2007; Ogura *et al.*, 2007). This suggests the existence of selective pressure that maintains the actin polymerization capabilities in EPEC and EHEC strains. Nonetheless, a fundamental question, which was the focus of this investigation, remains unanswered: what is the role of the Tir:Nck and Tir:IRTKS/IRSp53 pathways during infection *in vivo*?

## Results

### Construction of chromosomal *tir*_*CR*_ mutants

**Fig. 1:**
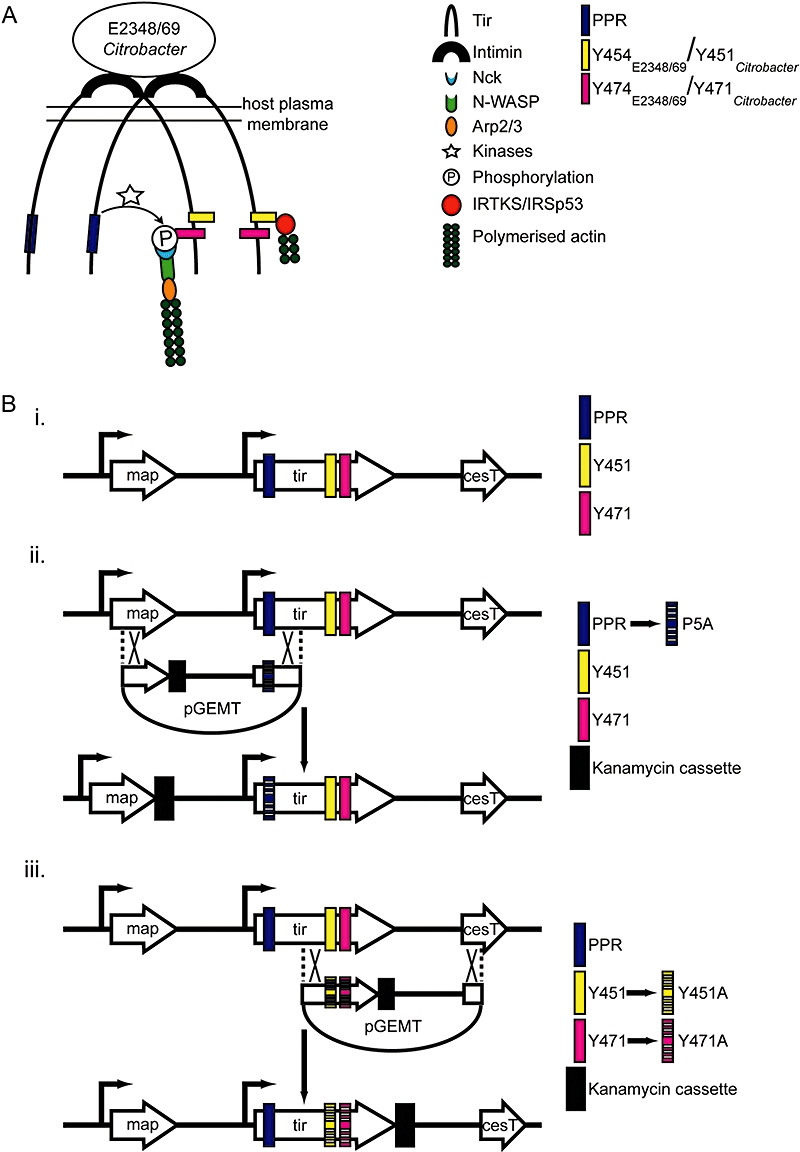
A. A diagram illustrating the Tir Y454/451 and Y474/471 actin polymerization pathways used by EPEC E2348/69 and *C. rodentium*. The polyproline region (PPR) of Tir EPEC is reported (Bommarius *et al.*, 2007) to recruit a number of protein tyrosine kinases that phosphorylate Y474 on adjacent Tir molecules and to maturate an Nck binding site, leading to activation of N-WASP and strong actin polymerization. Tir EPEC/CR can trigger inefficient actin polymerization via the NPY_454/1_-IRTKS/IRSp53 pathway. B. Schematic representation of the *tir*_*CR*_ chromosomal mutagenesis strategy. The genetic organization of the *map-tir-cesT* locus in wild-type *C. rodentium* is shown in (i). Recombinant pGEMT plasmids, containing *tir*_*CR*_ mutations were used as PCR templates and the amplified fragments, together with the lambda red recombinase, were used to introduce the mutations in tandem with an *aphT* kanamycin-resistance cassette into the endogenous *tir*_*CR*_ locus. Distinct constructs were used to introduce 5′-specific mock (Tir_N-ctrl_), Tir_P5A_ and Tir_1−33stop_ mutations (ii) or 3′-specific mock (Tir_C-ctrl_), Tir_Y451A_, Tir_Y471A_ and Tir_Y451A/Y471A_ mutations (iii).

We constructed *C. rodentium* mutants expressing Tir_P5A_ (mutated in the N-terminal polyproline sequence), Tir_Y451A_ (mutated in the IRSp53/IRTKS binding site), Tir_Y471A_ (mutated in the Nck binding site) and Tir_Y451A/Y471A_ (Fig. 1A). To this end, we developed a lambda red-based mutagenesis system (Datsenko and Wanner, 2000), involving insertion of a kanamycin cassette in the *map-tir* or *tir-cesT* intergenic regions, which allowed us to introduce site-directed *tir* mutations into the bacterial chromosome (Fig. 1B). Control mock mutants were generated by inserting kanamycin cassettes in the *map-tir* (N-terminal control – Tir_N-ctrl_) and *tir-cesT* (C-terminal control – Tir_C-ctrl_) intergenic regions without affecting the *tir* coding sequence. A nonsense mutant at Tir position 33 (Tir_1−33stop_) was used as a further control. Growth curves in minimal and rich media confirmed that the mutants and parental wild-type strains had identical growth rates (data not shown).

### Testing the Tir_CR_ mutants during infection of cultured cells

**Fig. 2:**
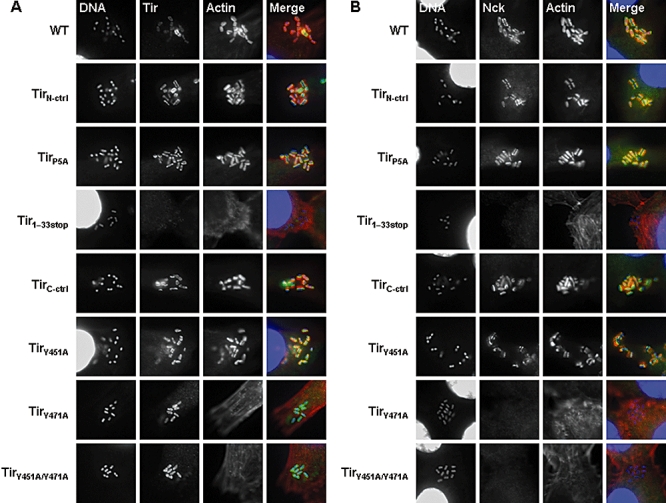
Infection of Swiss 3T3 cells with *C. rodentium*. Tir (A) was detected under adherent wild-type *C. rodentium* and *C. rodentium* expressing Tir_N-ctrl_, Tir_C-ctrl_, Tir_P5A_, Tir_Y451A_, Tir_Y471A_ and Tir_Y451A/Y471A_. Nck (B) and polymerized actin (A and B) were detected under wild-type *C. rodentium* and *C. rodentium* expressing Tir_N-ctrl_, Tir_C-ctrl_, Tir_P5A_ and Tir_Y451A_. No Nck or polymerized actin was detected under *C. rodentium* expressing Tir_Y471A_ or Tir_Y451A/Y471A_. Tir, Nck or polymerized actin were not detected under *C. rodentium* expressing Tir_1−33stop_.

We have recently shown that *C. rodentium* can efficiently adhere to and trigger actin polymerization in Swiss 3T3 fibroblast cells (Girard *et al.*, 2009a). Before investigating the effect of the mutagenesis *in vivo*, we characterized the Tir mutants *in vitro*. Infection of Swiss 3T3 cells showed recruitment of Tir, Nck and polymerized actin under adherent wild-type *C. rodentium* and *C. rodentium* expressing Tir_N-ctrl_, Tir_C-ctrl_, Tir_P5A_ and Tir_Y451A_ (Fig. 2). Focused Tir without detectable Nck or actin polymerization (Fig. 2) was detected under adherent *C. rodentium* expressing Tir_Y471A_ and Tir_Y451A/Y471A_, while neither focused Tir nor actin polymerization was detected under adherent *C. rodentium* expressing Tir_1−33stop_ (Fig. 2). These results show that the Tir polyproline sequence is dispensable for Nck recruitment and actin polymerization by *C. rodentium* both of which are dependent on Tir_Y471_.

### The role of the polyproline region and Tir residues Y451 and Y471 *in vivo*

**Fig. 3:**
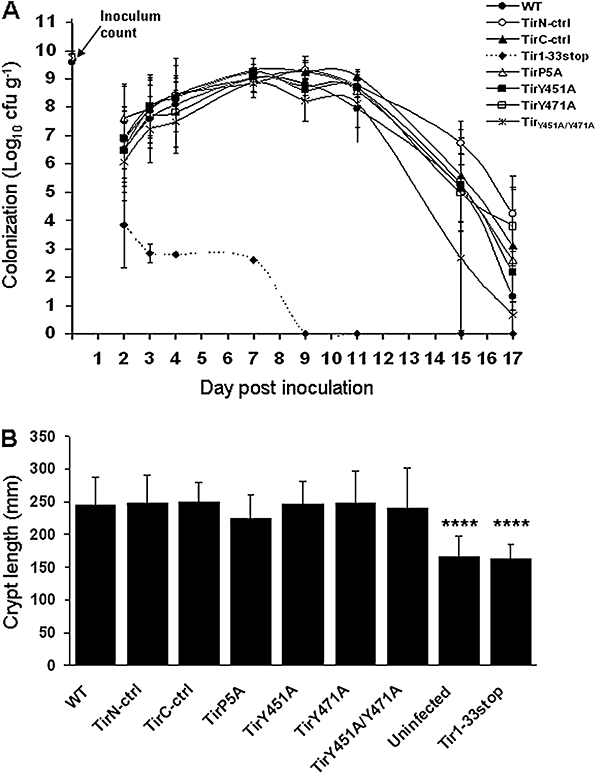
Colonization and clearance dynamics of *C. rodentium* strains after oral inoculation of C57Bl/6 mice. Mice inoculated with *C. rodentium* expressing Tir_P5A_, Tir_Y451A_, Tir_Y471A_ and Tir_Y451A/Y471A_ exhibit similar colonization dynamics (A) and crypt hyperplasia, measured at day 8 post inoculation (B), as wild-type *C. rodentium* or *C. rodentium* expressing Tir_N-ctrl_, Tir_C-ctrl_. Both colonization and hyperplasia were significantly different from uninfected mice or mice infected with *C. rodentium* expressing Tir_1−33stop_; ****P < 0.0001. The inoculum count is the number of viable bacteria in 200 μl used to inoculate mice by oral gavage. Data are represented as mean ± SD.

We investigated the impact of Tir mutagenesis on colonization and persistence of *C. rodentium in vivo* by enumerating colony-forming units per gram of stools (cfu g^−1^) collected at regular intervals following oral inoculation of C57BL/6 mice. This has shown that inoculation with *C. rodentium* expressing Tir_P5A_, Tir_Y451A_, Tir_Y471A_ and Tir_Y451A/Y471A_ (Fig. 3A) resulted in the same colonization dynamics as wild-type *C. rodentium* or the control strains expressing Tir_N-ctrl_ and Tir_C-ctrl_ (Fig. 3A); infection with all strains peaked at day 7 and started to clear from day 15 post inoculation. *C. rodentium* expressing Tir_1−33stop_ was rapidly cleared and failed to initiate an infection. *C. rodentium* expressing Tir_P5A_, Tir_Y451A_, Tir_Y471A_ and Tir_Y451A/Y471A_ (Fig. 3B) were as efficient in triggering colonic hyperplasia as wild-type *C. rodentium* and *C. rodentium* expressing Tir_N-ctrl_ and Tir_C-ctrl_ (Fig. 3B); crypt length was significantly higher than in mice infected with *C. rodentium* expressing Tir_1−33stop_ or uninfected mice (Fig. 3B).

### Recruitment of adaptor and signalling molecules to the site of *C. rodentium* attachment *in vivo*

**Fig. 4:**
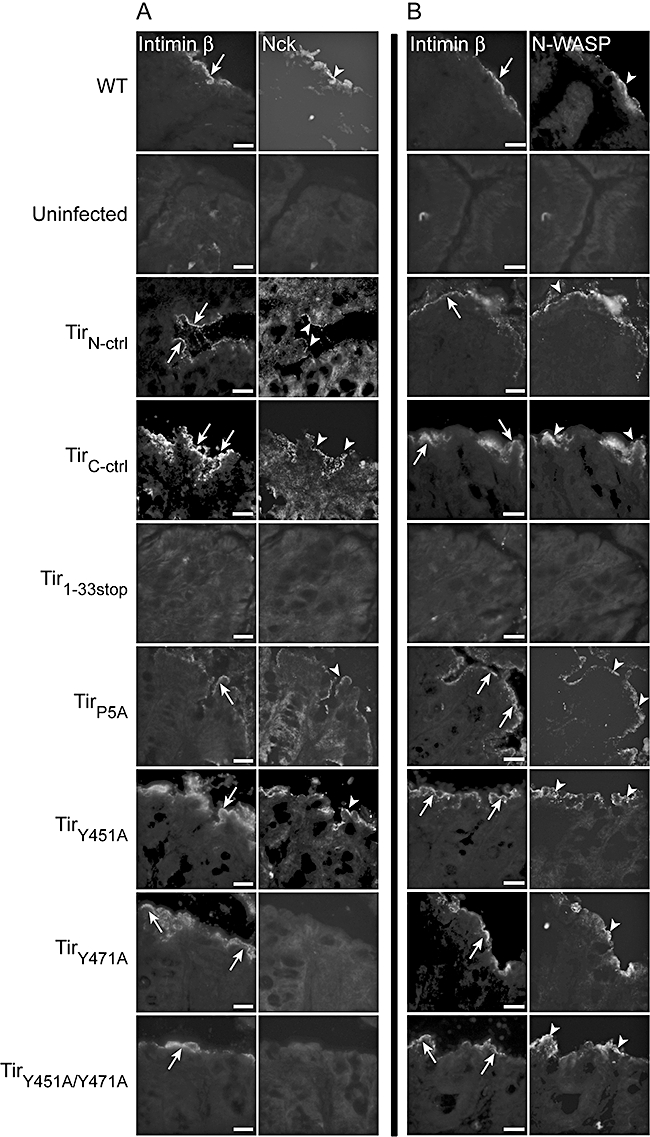
Recruitment of Nck and N-WASP to the *C. rodentium* attachment sites *in vivo*. A. Nck (arrowheads) is recruited under adherent wild-type *C. rodentium* (arrows) and *C. rodentium* expressing Tir_N-ctrl_, Tir_C-ctrl_, Tir_P5A_ and Tir_Y451A_. Nck was not detected under intimately adherent *C. rodentium* expressing Tir_Y471A_ or Tir_Y451A/Y471A_. B. N-WASP (arrowheads) is recruited *in vivo* under intimately adherent wild-type *C. rodentium* (arrows) and *C. rodentium* expressing Tir_N-ctrl_, Tir_C-ctrl_, Tir_P5A,_ Tir_Y451A_, Tir_Y471A_ and Tir_Y451/Y471A_. Neither intimately adherent bacteria, nor Nck or N-WASP recruitment was observed on sections derived from mice inoculated with *C. rodentium* expressing Tir_1−33stop_ or uninfected mice. Bacteria were labelled with anti-intimin β. Bar = 20 μm.

We first processed thin colonic sections to determine if Nck is recruited under adherent *C. rodentium in vivo.* Nck was detected in mice infected with wild-type *C. rodentium* and *C. rodentium* expressing Tir_N-ctrl_, Tir_C-ctrl_, Tir_P5A_ and Tir_Y451A_, while no Nck recruitment was detected under adherent *C. rodentium* expressing Tir_Y471A_, Tir_Y451A/Y471A_ and Tir_1−33stop_ (Fig. 4A).

**Fig. 5:**
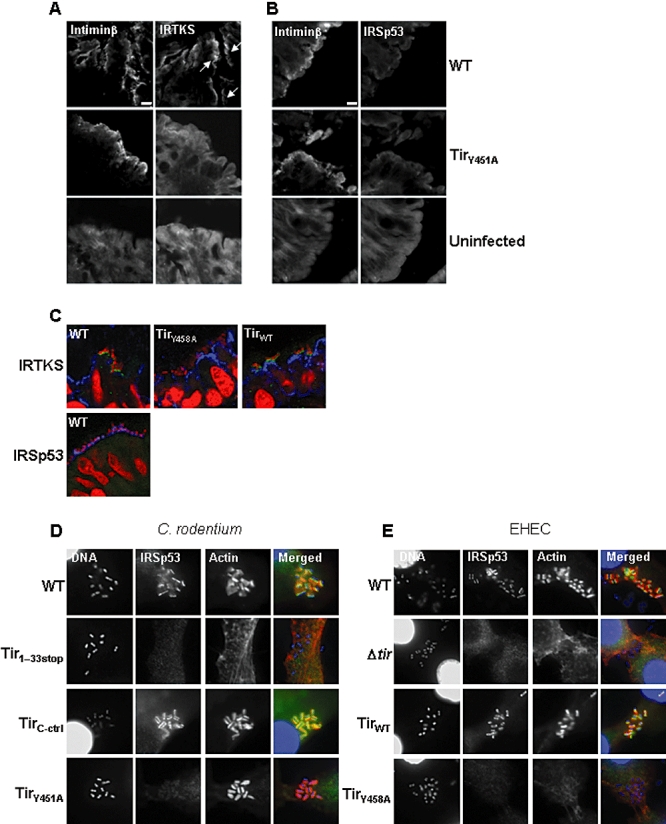
Recruitment of IRTKS (A) and IRSp53 (B) to the *C. rodentium* attachment sites *in vivo*. IRTKS (arrows) is recruited under adherent wild-type *C. rodentium*. IRTKS was not detected under intimately adherent *C. rodentium* expressing Tir_Y451A_ or on uninfected tissues. IRSp53 was not detected in either the infected or uninfected tissues. Bar = 20 μm. (C) Immunofluorescence staining of human terminal ileal mucosa infected with wild-type EHEC, EHEC Δ*tir*(pICC422), expressing Tir_Y458A_ and EHEC Δ*tir*(pICC421), expressing Tir_wt_. IRTKS (green) was detected under adherent wild-type EHEC and EHEC Δ*tir* expressing Tir_wt_ but not under adherent EHEC Δ*tir* expressing Tir_Y458A_. No IRSp53 staining was observed under attached wild-type EHEC bacteria. Tir was stained in blue (lower panel) to facilitate detection of A/E lesions. Sections were counterstained with anti-cytokeratin (blue, upper panels) and propidium iodide (red) to visualize epithelial cells and cell nuclei/bacteria respectively. Shown are merged images of all fluorescence channels. Infection of Swiss 3T3 cells with *C. rodentium* (D) or EHEC (E) strains. IRSp53 was detected under adherent wild-type *C. rodentium*, *C. rodentium* expressing Tir_C-ctrl_, wild-type EHEC and EHEC Δ*tir* expressing Tir_WT_. IRSp53 was not detected under *C. rodentium* expressing Tir_Y451A_ or Tir_1−33stop_ and under EHEC Δ*tir* expressing Tir_Y458A_ or EHEC Δ*tir*.

We next investigated recruitment of IRSp53 and IRTKS to adherent *C. rodentium*. While IRTKS was recruited to the site of adherent wild-type *C. rodentium* (Fig. 5A), IRSp53 was undetectable ((using an IRSp53 antiserum made against a 21-amino-acid peptide that is conserved between the human and the mouse IRSp53) (Weiss *et al.*, 2009)) (Fig. 5B). IRTKS was also detected at the attachment sites of *C. rodentium* expressing Tir_N-ctrl_, Tir_C-ctrl_, Tir_P5A_ and Tir_Y471A_ (data not shown) but was not detected under adherent *C. rodentium* expressing Tir_Y451A/Y471_, Tir_1−33stop_ (data not shown), Tir_Y451A_ or on uninfected tissues (Fig. 5A). These results show that IRTKS is recruited in the mouse gut mucosa beneath adherent *C. rodentium* in a NPY motif-dependent manner, while IRSp53 does not seem to be recruited under adherent bacteria in the *in vivo* mouse model.

In order to support our conclusions using an alternative model, we tested the recruitment of IRSp53 and IRTKS to the site of EHEC O157:H7 (strain TUV 93-0) attachment on human ileal *in vitro* organ culture (IVOC). Similarly to the results of *C. rodentium* infection, IRTKS, but not IRSp53, was detected at the bacterial attachment sites in a Tir_EHEC_ Y458-dependent manner (Fig. 5C).

As we were unable to observe IRSp53 recruitment under adherent bacteria either during *C. rodentium* mouse infection or in EHEC-infected human IVOC, we controlled our ability to detect IRSp53 recruitment to bacterial attachment sites *in vitro*. Consistent with the published data (Weiss *et al.*, 2009) we found that IRSp53 was recruited at the site of *C. rodentium* and EHEC adhesion on Swiss 3T3 cells in a NPY motif-dependent manner (Fig. 5D and E). Recruitment of IRSp53 was also detected by immunofluorescence in infected HeLa cells (data not shown). Therefore, our inability to detect recruitment of IRSp53 to *C. rodentium* and EHEC adhering to mucosal surfaces might be due to the fact that the protein is either not expressed in these tissues or the expression level is below the detection sensitivity.

Finally we investigated recruitment of N-WASP *in vivo* following *C. rodentium* infection. Immunofluorescent staining revealed recruitment to *C. rodentium* attachment sites independently of the polyproline sequence and Tir residues Y451 and Y471 as N-WASP was detected in mice inoculated with any of the site-directed mutants except *C. rodentium* expressing Tir_1−33stop_ (Fig. 4B). These results show that N-WASP is recruited *in vivo* to the bacterial attachment sites by a novel mechanism independent of the Tir:Nck and Tir:IRTKS complexes.

### Formation of A/E lesions by the different Tir mutants *in vivo*

**Fig. 6:**
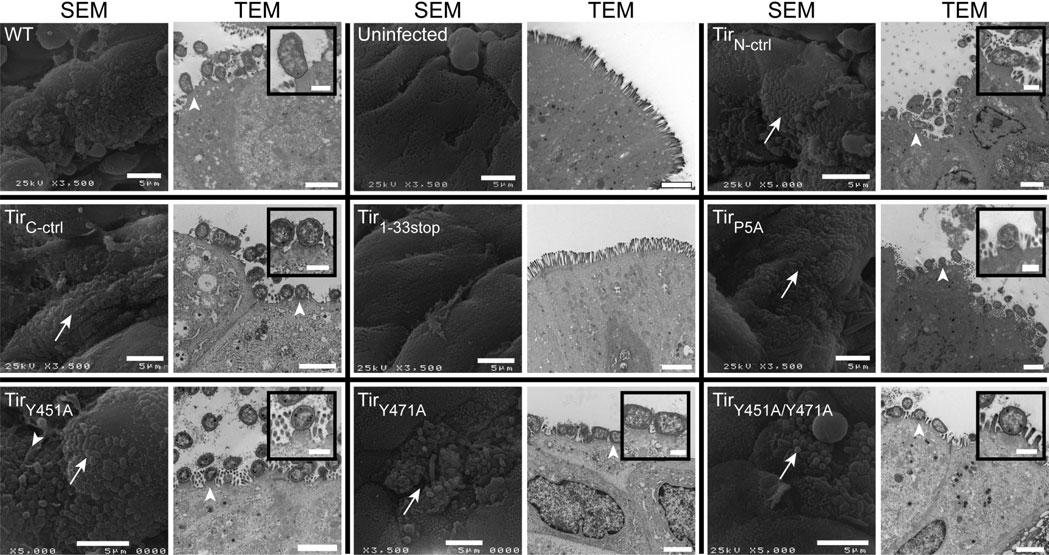
Scanning and transmission electron microscopy of mice colonic epithelium infected with wild-type *C. rodentium* and *C. rodentium* expressing Tir_N-ctrl_, Tir_C-ctrl_, Tir_P5A_, Tir_Y451A_, Tir_Y471A_, Tir_Y451/Y471A_. Local effacement of the brush border microvilli, intimately adherent bacteria (arrow) and accumulation of electron-dense material (arrowheads) were observed following inoculation of mice with any of the *C. rodentium* strains. Intact brush border microvilli were observed following mice infection with *C. rodentium* expressing Tir_1−33stop_ or on uninfected mice. Tissues were collected at day 8 post inoculation. Bar = 5 μm (SEM), 2 μm (TEM) or 500 nm (TEM, insets).

The data presented thus far show that assembly of the Tir:Nck and Tir:IRTKS complexes by *C. rodentium in vivo* is dependent on Y471 and Y451 residues respectively, but independent of the polyproline N-terminal sequence. We next investigated the role of these Tir residues in A/E lesion formation. Transmission and scanning electron microscopy revealed typical A/E lesions on colonic mucosa in mice inoculated with wild-type *C. rodentium* and *C. rodentium* expressing Tir_N-ctrl_, Tir_C-ctrl_, Tir_P5A_, Tir_Y451A_, Tir_Y471A_ and Tir_Y451A/Y471A_ (Fig. 6). No morphological differences or variation in the overall level of electron-dense material was seen at the site of attachment of any of the *C. rodentium* strains. No A/E lesions were detected in mice infected with *C. rodentium* expressing Tir_1−33stop_. These results show that, while Nck and IRTKS are recruited to *C. rodentium* attachment sites in mice, their recruitment is not essential for A/E lesion formation.

### *C. rodentium* expressing Tir_Y451A_, Tir_471A_ and Tir_Y451A/Y471A_ are out-competed by the wild-type strain during mixed infections

**Fig. 7:**
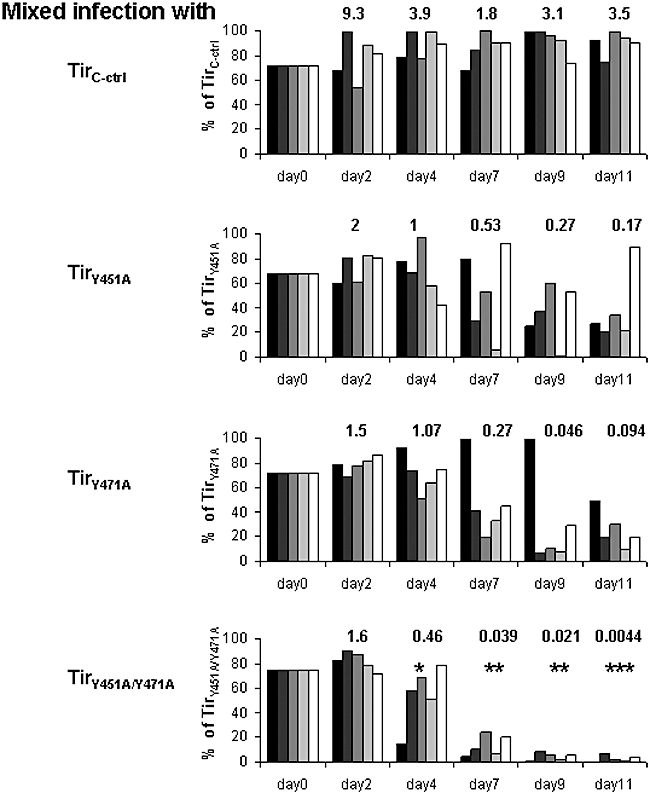
*C. rodentium* expressing Tir_C-ctrl_, Tir_Y451A_, Tir_Y471A_ and Tir_Y451A/Y471A_ (test strains) were compared in mixed infection experiments with wild-type (WT, reference) strain. The ratio between the test and the reference strains was determined in percentage on days 2, 4, 7, 9 and 11 post inoculation in each of the five mice per group (each column represents individual mouse). The CI values were calculated and the median CI is indicated on the graph. The asterisks indicate that using global statistical analysis the CI of Tir_Y451A/Y471A_ versus WT is significantly lower than the CI of Tir_C-ctrl_ versus WT. *P < 0.05, **P < 0.01 and ***P < 0.001.

As none of the Tir mutations, but Tir_1−33stop_, affected bacterial load or A/E lesion formation, we compared their competitiveness against wild-type *C. rodentium in vivo*. To this end, we conducted mixed infection experiments in which groups of five C57BL/6 mice were inoculated at a ratio of approximately 2:1 test strain (*C. rodentium* expressing Tir_C-ctrl_, Tir_Y451A_, Tir_Y471A_ or Tir_Y451A/Y471A_) to a reference strain (wild-type *C. rodentium*). At days 2, 4, 7, 9 and 11 post inoculation the ratio between the two populations (test strain versus reference strain) and the competitive index (CI) were calculated for each group (Fig. 7). In order to neutralize any potential negative effects of the chromosomal kanamycin cassette insertion on the *in vivo* fitness of the mutants, the CI of the mutant strains was directly compared with the CI of the mock mutant control. Generally, a test strain with a CI of less than 0.5 is considered attenuated, whereas a CI equal or higher than 1 indicates that the test strain colonizes at least as well as the reference strain (Mundy *et al.*, 2004).

We conducted pairwise and global statistical analysis of the CI of *C. rodentium* expressing Tir_Y451A_, Tir_Y471A_ and Tir_Y451A/Y471A_ compared with the CI of *C. rodentium* expressing Tir_C-ctrl_. We also compared the CI of *C. rodentium* expressing Tir_P5A_ with the CI of *C. rodentium* expressing Tir_N-ctrl_. While *C. rodentium* expressing Tir_P5A_ was as competitive as the wild-type strain (data not shown), pairwise analysis showed that from day 9 post inoculation *C. rodentium* expressing Tir_Y451A_ and Tir_Y471A_ were significantly out-competed compared with *C. rodentium* expressing Tir_C-ctrl_. *C. rodentium* expressing Tir_Y451A/Y471A_ showed a greater degree of attenuation, which was seen as early as day 4 post inoculation. Global statistical analysis of the CI of *C. rodentium* expressing Tir_Y451A_, Tir_Y471A_ and Tir_Y451A/Y471A_ showed that from day 4 post inoculation, *C. rodentium* expressing Tir_Y451A/Y471A_ strain was highly attenuated compared with *C. rodentium* expressing Tir_C-ctrl_, with a CI of 0.0044 compared with 3.5 for the control strain at day 11 (Fig. 7). These results show that although not affecting bacterial load and A/E lesion formation *in vivo*, Tir residues Y451 and Y471, and by extension Tir:Nck and Tir:IRTKS complexes, provide the bacterium with competitive edge and higher fitness *in vivo*.

## Discussion

The Nck binding site and the NPY motif within Tir are conserved among typical EPEC, non-O157 EHEC and *C. rodentium* (reviewed in Garmendia *et al.*, 2005; Frankel and Phillips, 2008). The absence of the Nck binding site in Tir EHEC O157:H7 is compensated by expression of TccP/EspF_U_ (Campellone *et al.*, 2004b; Garmendia *et al.*, 2004), which similarly to Nck, activates N-WASP (Garmendia *et al.*, 2006; Campellone *et al.*, 2008; Cheng *et al.*, 2008; Sallee *et al.*, 2008). This suggests the existence of selective pressure for the preservation of these signalling pathways. However, despite intense investigations, the role of these signalling pathways *in vivo* is not fully understood.

In this study we investigated the role of Tir tyrosine residues Y451 and Y471 during the entire infection cycle using the *C. rodentium* model. After confirming that *C. rodentium* expressing Tir_Y451A_, Tir_Y471A_ and Tir_Y451A/Y471A_ had the same *in vitro* phenotypes as their corresponding EPEC Y454 and Y474 Tir mutants (in terms of actin polymerization and Nck recruitment), we investigated their phenotype *in vivo*. We found that *C. rodentium* expressing the single Tir_Y451A_ and Tir_Y471A_ substitutions and the double Tir_Y451A/Y471A_ mutation exhibited colonization dynamic, pathogen load, tissue targeting, pathology (i.e. colonic hyperplasia) and A/E lesion formation abilities that were indistinguishable from the parental wild-type strain. Importantly, we found that even though *C. rodentium* expressing Tir_Y471A_ lost the ability to recruit Nck, N-WASP was still found recruited under attached bacteria *in vivo*. *C. rodentium* expressing Tir_Y451A_ recruited both Nck and N-WASP to the site of bacterial attachment *in vivo*. In addition, A/E lesions induced by *C. rodentium* expressing any of the Tir mutants, but Tir_1−33stop_, showed similar accumulation of electron-dense material under adherent bacteria.

Consistent with our data, Deng *et al.* (2003) have previously shown that *C. rodentium* expressing Tir_Y471F_ induced typical A/E lesions *in vivo* at day 8 post oral challenge. However, as these studies were done using plasmid-encoded Tir, the level of colonization of the *tir* mutant was greatly lower than that achieved by the wild-type strain. Using the human IVOC model, Schuller *et al.* (2007) have shown that while Tir is essential for colonization of the human gut mucosa, mutation in Y474 of EPEC E2348/69 Tir abolished Nck recruitment but did not affect the ability of the bacteria to recruit N-WASP and to induce typical A/E lesions. Moreover, and consistent with our *C. rodentium* data, EPEC E2348/69 expressing Tir_Y454F/Y474F_ still recruited N-WASP to the bacterial attachment sites on human IVOC. When considered together these data suggest that actin polymerization on mucosal surfaces is triggered by EPEC and *C. rodentium* independently of Y454/1 and Y474/1 residues by a yet unidentified mechanism.

Recent reports have shown that Tir recruits IRTKS (Vingadassalom *et al.*, 2009) and/or IRSp53 (Weiss *et al.*, 2009) in an NPY-dependent manner. Therefore, we investigated if IRTKS or IRSp53 are recruited to the *C. rodentium* attachment sites *in vivo*. IRTKS was detected under attached bacteria and its recruitment was Y451-dependent. In contrast, no IRSp53 was detected under adherent bacteria *in vivo*. Similarly we found that IRTKS, but not IRSp53, was recruited under adherent EHEC bacteria using human IVOC.

Adhesion of EHEC O157 to human (Garmendia *et al.*, 2004) and bovine (Girard *et al.*, 2009b) IVOC and bovine ileal loops (Vlisidou *et al.*, 2006) was reported to be Tir-dependent but independent of the Tir-IRTKS-TccP/EspF_U_ complex, which is consistent with our observation that EHEC adhered to human IVOC independently of IRTKS recruitment. *In vivo* calf studies revealed no measurable differences in colonization levels between wild type and EHEC O157 Δ*tccP/espF*_*U*_ (Vlisidou *et al.*, 2006). However, in infant rabbits colonization efficiency of the *tccP/espF*_*U*_ EHEC O157 mutant was similar to the parent strain in the ileum but was reduced in the large bowel at 7 days post infection (Ritchie *et al.*, 2008). In gnotobiotic piglets, expression of TccP/EspF_U_ was associated with larger-size adherent bacterial aggregates (Ritchie *et al.*, 2008). Together, these results are consistent with the notion that the Tir NPY signalling pathway is not essential for the formation of A/E lesions and for the establishment of colonization, but promotes bacterial expansion from the initial infection sites.

In addition to the Tir Y451 and Y471 pathways, we investigated the role of the Tir N-terminal polyproline sequence as it was reported to play a role in the recruitment of kinases involved in Tir phosphorylation, Nck recruitment and actin polymerization. Contrary to its reported role in EPEC (Bommarius *et al.*, 2007), the polyproline sequence plays no role in recruitment of Nck and actin polymerization in *C. rodentium in vitro* and *in vivo*. These data are consistent with a previous report showing that deletion of the entire N-terminus of EPEC and EHEC Tir did not inhibit actin polymerization (Campellone *et al.*, 2004a; 2006).

Taken together, our results show that while Tir Y471 recruits Nck and Y451 recruits IRTKS, these signalling pathways are dispensable for colonization, colonic hyperplasia and A/E lesion formation. This conclusion raises a fundamental question: does the ability to activate these pathways benefit the bacterium? In order to address this experimentally, we performed mixed infection studies. By comparing the CIs of *C. rodentium* expressing mock or *tir* mutants, we have shown that in mixed infections of wild type with either of the single Y451 and Y471 Tir mutants the latter strains were similarly out-competed. Importantly, mixed infections of wild type and *C. rodentium* expressing the double Tir_Y451A/Y471A_ mutant resulted in rapid decline of the mutant. These results suggest that Tir_Y451A_ and Tir_Y471A_ contribute independently to *C. rodentium* competitiveness *in vivo*. The fact that *C. rodentium* expressing Tir_Y451A/471A_ is significantly more attenuated than each of the single mutants alone suggests a cooperative (accumulative) function of the Y451 and Y471 pathways. We conclude that Tir residues Y454/1 and Y474/1 contribute to *in vivo* competitiveness, probably during mixed infections.

In conclusion, our results show that despite defining EPEC, EHEC and *C. rodentium* infection, we lack basic knowledge of the mechanisms involved in A/E lesion formation. Moreover, we have shown that recruitment of N-WASP to the site of bacterial attachment *in vivo* occurs independently of Tir residues Y451 and Y471. While IRTKS is recruited to bacterial adhesion sites *ex vivo* and *in vivo*, it is not essential for A/E lesion formation or recruitment of N-WASP. Finally, our results show that although not involved in A/E lesion formation Tir residues Y451 and Y471 play an important role in pathogen host interaction.

## Experimental procedures

### Bacterial strains and growth conditions

The bacterial strains, plasmids and primers used in this study are listed in Table 1. Bacteria were grown in Luria–Bertani (LB) medium, M9 minimum media (Mundy *et al.*, 2004) or in Dulbecco's modified Eagle's medium (DMEM) supplemented with kanamycin (50 mg ml^−1^), ampicilin (100 mg ml^−1^) and nalidixic acid (50 mg ml^−1^) as required.

**Table 1:** Strains, plasmids and primers used in this study.

	Description	Reference
Strains		
ICC169	Wild type *C. rodentium* (serogroup O152); Nal^R^	Wiles *et al.* (2004)
ICC293	*C. rodentium* expressing Tir_N-ctrl_	This study
ICC294	*C. rodentium* expressing Tir_C-ctrl_	This study
ICC295	*C. rodentium* expressing Tir_1−33stop_	This study
ICC296	*C. rodentium* expressing Tir_P5A_	This study
ICC297	*C. rodentium* expressing Tir_Y451A_	This study
ICC298	*C. rodentium* expressing Tir_Y471A_	This study
ICC301	*C. rodentium* expressing Tir_Y451/471A_	This study
TUV 93-0	EDL933, EHEC O157:H7, *stx*1 and *stx*2 mutant	Riley *et al.* (1983)
KC5	TUV 93-0 *tir* deletion mutant	Murphy and Campellone (2003)
Plasmids		
pSA10	pKK177-3 containing *lacI* gene	Schlosser-Silverman *et al.* (2000)
pICC421	pSA10 derivative encoding EHEC Tir	Mousnier *et al.* (2008)
pICC422	pICC421 derivative encoding EHEC Tir_Y458A_	Mousnier *et al.* (2008)
pET28a	Expressing vector	Novagen
pGEMT	Cloning vector	Promega
pKD46	Coding the lambda Red recombinase	Datsenko and Wanner (2000)
pSB315	A plasmid coding for the kanamycin resistance *aphT* cassette	Galan *et al.* (1992)
pICC431	pET28a expressing N-terminal His-tagged EVHI domain of N-WASP	Girard *et al.* (2009a)
pICC432	pGEMT vector containing the 3′ end of *map* (bp 377–612), the *aphT* cassette, *map-tir* intergenic region and the 5′ end of *tir*_*CR*_ (bp 1–331)	This study
pICC433	pGEMT vector containing the 3′ end of *tir*_*CR*_ (bp 1067−1644), the *aphT* cassette, *tir-cesT* intergenic region and the 5′ end of *cesT* (bp 1–388)	This study
pICC434	pICC432 containing a stop codon at amino acid position 33 of Tir_CR_	This study
pICC435	pICC432 containing the *tir*_*CR*_ P5A mutation	This study
pICC436	pICC433 containing the *tir*_*CR*_ Y451A mutation	This study
pICC437	pICC433 containing the *tir*_*CR*_ Y471A mutation	This study
pICC438	pICC433 containing the *tir*_*CR*_ Y451A/Y471A mutation	This study
Primer name	Nucleotide sequence (restriction site in bold)	
C*map*-Fw	5′-gtgcacaatcatcaatcagtcac-3′	
EcoRI-C*map*-Rv	5′-ccgg**aattc**ctacagcctggtatcctgcac-3′	
EcoRI-[*map*-*tir*]-Fw	5′-ccg**gaattc**gagggtattttgggcttaatgg-3′	
N*tir*-Rv	5′-ggaattccatatgcaacacgaaatacagaggatcc-3′	
C*tir*-Fw	5′-catgccatggtggatctctcatcaggtattgg-3′	
EcoRI-C*tir*-Rv	5′-ccg**gaattc**ttagacgaaacgttcaactccc-3′	
EcoRI-[*tir*-*cesT*]-Fw	5′-ccg**gaattc**atatataatgggtattttgttggggggg-3′	
N*cesT*-Rv	5′-gattatgtaataccaggtacagg-3′	
TirP5A-Fw	5′-gcagccctagcatcacaaacagacggcgcgacaag-3′	
TirP5A-Rv	5′-cgctgctgcaattaaattgttacttatattattattaccaag-3′	
TirY451A-Fw	5′-gctgaaggttggatgtccagg-3′	
TirY451A-Rv	5′-ggctggattcaccacatcgccagag-3′	
TirY471A-Fw	5′-gatgaagtcgctccggatcct-3′	
TirY471A-Rv	5′-ggctataggctcttctggagcgag-3′	

### Introduction of site-directed *tir*_*CR*_ mutants into the bacterial chromosome

The *C. rodentium* expressing mutated Tir were generated using a lambda red-based mutagenesis system (Datsenko and Wanner, 2000). We introduced site-directed *tir* mutations into the endogenous chromosomal *tir* gene, together with a kanamycin cassette, either in the *map-tir* or the *tir-cesT* intergenic regions for 5′ and 3′ mutagenesis respectively.

In order to mutate the N-terminal polyproline region we cloned the 3′ end of *map* (base pairs 377–612) followed by a non-polar *aphT* cassette (Galan *et al.*, 1992), which confers kanamycin resistance, the *map-tir* intergenic region and the 5′ end of *tir*_*CR*_ (base pairs 1–331) into a pGEMT vector. The 3′ end of *map* was amplified using primer pair C*map*-Fw and EcoRI-C*map*-Rv and the *map-tir* intergenic region with the 5′ end of *tir*_*CR*_ was amplified using primer pair and EcoRI-[*map-tir*]-Fw, N*tir*-Rv. The two fragments were digested with EcoRI, ligated to each together and then cloned into pGEMT. The non-polar *aphT* cassette was then inserted into the EcoRI site between the two fragments and the orientation of the kanamycin cassette checked by PCR. The plasmid, named pICC432, was used to generate the control *C. rodentium* mutant expressing Tir_N-ctrl_, as described below.

Similarly, in order to mutate the C-terminal tyrosine residues we cloned the 3′ end of *tir*_*CR*_ (base pairs 1067−1644), followed by a non-polar *aphT* cassette, the *tir-cesT* intergenic region and the 5′ end of *cesT (*base pairs 1–388) into pGEMT.

The 3′ end of *tir*_*CR*_ was amplified using primer pair C*tir*-Fw and EcoRI-C*tir*-Rv and the *tir-cesT* intergenic region with the 5′ end of *cesT* was amplified using the primer pair EcoRI-[*tir-cesT*]-Fw and N*cesT*-Rv. The two fragments were digested with EcoRI, ligated to each other, cloned into pGEMT and the non-polar *aphT* cassette was inserted into the EcoRI site as described above, generating plasmid pICC433, which was used to generate the control *C. rodentium* mutant expressing Tir_C-ctrl_.

Tir_CR_ mutagenesis was then carried out by inverse-PCR pICC432 and pICC433 as templates for 5′ and 3′ site-directed mutagenesis respectively. The primer pair TirP5A-Fw and TirP5A-Rv was used to mutate the five proline residues of *tir*_*CR*_ polyproline region into alanines (Tir_P5A_), generating pICC435. Similarly, the primer pair TirY451A-Fw and TirY451A-Rv and TirY471A-Fw and TirY471A-Rv were used to mutate the tyrosine residues Y451 and Y471 into alanines (Tir_Y451A_ and Tir_Y471A_) generating pICC436 and pICC437 respectively. The double tyrosine mutant (Tir_Y451A/Y471A_) was generated by superimposing Y471A on the Y451A mutant, generating pICC438. All pGEMT derivatives were checked by DNA sequencing using an automated DNA sequencer (ABI 377). While sequencing the putative Tir_P5A_ mutants we identified a non-specific mutant containing a frameshift that created a stop codon at Tir amino acid position 33 (Tir_1−33stop_). This clone, named pICC434, was used as a nonsense mutant control.

To introduce *tir*_*N-ctrl*_, *tir*_*1−33stop*_ and *tir*_*P5A*_ site-directed mutants into *C. rodentium* chromosome, the inserts in pICC432, pICC434 and pICC435 were PCR-amplified using the primer pair C*map*-Fw and N*tir*-Rv. Similarly, to introduce *tir*_*C-ctrl*_, *tir*_*Y451A*_, *tir*_*Y471A*_ and *tir*_*Y451A/Y471A*_ site-directed mutants into *C. rodentium* chromosome, the inserts in pICC433, pICC436, pICC437 and pICC438 were amplified using the primer pair C*tir*-Fw and N*cest*-Rv. The PCR products were electroporated into wild-type *C. rodentium* (ICC169) containing pKD46 encoding the lambda Red recombinase (Datsenko and Wanner, 2000). Transformants were selected on kanamycin plates and the insertion of site-directed *tir*_*CR*_ mutants into *C. rodentium* chromosome was confirmed by PCR and DNA sequencing.

### Cell culture

Swiss 3T3 cell line was grown in DMEM containing 4500 mg l^−1^ glucose and supplemented with 10% fetal calf serum and 2 mM glutamine at 37°C in 5% CO_2_. Cells were seeded onto glass coverslips in 24-well plates at a density of 5 × 10^4^ cells per well, 48 h before infection. EHEC (Mousnier *et al.*, 2008) or *C. rodentium* (Girard *et al.*, 2009a) strains used for *in vitro* assays were grown for 8 h in LB broth, then transferred into fresh, sterile DMEM containing 1000 mg l^−1^ glucose and incubated static at 37°C in 5% CO_2_ overnight prior to infection. Each coverslip was infected with 25 μl of the appropriate overnight culture, centrifuged at 1000 r.p.m. for 5 min at room temperature, and then incubated at 37°C in 5% CO_2_ for 5 h. The cell culture medium was renewed half way through the infection period. After washes with phosphate-buffered saline (PBS), infected cells were fixed for 20 min in 4% paraformaldehyde, permeabilized with 0.1% Triton for 5 min and labelled by indirect immunofluorescence, using rabbit anti-Tir_EHEC_ (1:500) (Batchelor *et al.*, 2004; Girard *et al.*, 2007), rabbit anti-Nck (1:300) [Millipore (Upstate), Lake Placid, NY, USA] or rabbit anti-IRSp53 (1:50) (Weiss *et al.*, 2009) and carbocyanine-2-conjugated donkey anti-rabbit IgG (Jackson ImmunoResearch Europe, Soham, Cambridgeshire, UK) secondary antibodies were used. Phalloidin-Tetramethyl Rhodamine Iso-Thiocyanate (Sigma) was used to stain F-actin, while bacterial DNA was counterstained with Hoechst 33342. Coverslips were mounted with Pro-Long Gold antifade reagent (Invitrogen) and analysed using an Axio Imager M1 microscope (Carl Zeiss MicroImaging GmbH, Germany). Images were acquired using an AxioCam MRm monochrome camera and deconvoluted using AxioVision (Carl Zeiss MicroImaging GmbH, Germany),

### Mice

Pathogen-free female 18–20 g C57Bl/6 mice were purchased from Charles River. All animals were housed in individually HEPA-filtered cages with sterile bedding and free access to sterilized food and water. All animal experiments were performed in accordance with the Animals Scientific Procedures (Act 1986) and were approved by the local Ethical Review Committee. Independent single infection experiments were performed twice using four to eight mice per group. Mice inoculated with mock mutant and nonsense mutant strains were included in every experiment. Mice inoculated with wild-type strain and uninfected mice were included in parallel with mutant strains.

### Oral infection of mice

For single infection experiments, mice were inoculated by oral gavage with 200 μl of overnight LB-grown *C. rodentium* suspension in PBS (≈ 5 × 10^9^ cfu). The number of viable bacteria used as inoculum was determined by retrospective plating onto LB agar containing antibiotics. Stool samples were recovered aseptically at various time points after inoculation and the number of viable bacteria per gram of stool was determined by plating onto LB agar (Wiles *et al.*, 2005). At day 8 post inoculum, the mice were culled and the colonic tissues were collected for histopathological and microscopic studies as described below.

For mixed infection experiments, the two overnight LB-grown bacterial strains to be competed against each other *in vivo* were combined in a ratio of 1:1 (approximately 2 × 10^9^ cfu for each strain) in 200 μl PBS and used to infect mice by oral gavage. Dilutions of the inoculum were plated on respective antibiotic-containing plates to determine the precise ratio of the two bacterial strains (test strain/reference strain). The ratio of viable bacteria determined in our inoculum was of approximately 2:1 for all CI experiments. Stool samples were collected at regular intervals and the CI was calculated by dividing the ratio of test strain cfu and reference strain cfu from the stools by the ratio of test strain cfu to reference strain cfu in the inoculum (Mundy *et al.*, 2003). The CI experiments were carried out using five animals per group and the CI was determined at days 2, 4, 7, 9 and 11 post inoculation.

### Harvesting, collection of samples and histopathology

Segments of the terminal colon of each mouse were collected post mortem at day 8 post inoculation, rinsed of their content and fixed in 10% buffered formalin for microscopic examination. Formalin-fixed tissues were then processed, paraffin-embedded, sectioned at 5 μm, and stained with haematoxylin and eosin (HandE) according to standard techniques. Formalin-fixed, paraffin-embedded sections (FFPE) were examined by light microscopy for the presence of intimately adhering bacteria on intestinal cells, as previously described (Girard *et al.*, 2005b). Crypts length was also evaluated and the length of at least six well-oriented crypts has been measured on each section.

Additional colonic segments were fixed in 2.5% glutaraldehyde for further electron microscopy analysis, while some were embedded in optimal cutting temperature medium (Raymond A Lamb Limited, UK), then snap-frozen in liquid nitrogen for further cryosectioning.

### IVOC and immunofluorescence staining of cryosections

Human IVOC, cryosectioning and immunofluorescence staining were performed as described previously (Schuller *et al.*, 2007) with ethical approval and informed consent. Biopsies from the terminal ileum were infected with wild-type EHEC (TUV 93-0), EHEC Δ*tir* (KC5) strain expressing EHEC Tir_WT_ (pICC421) or Tir_Y458A_ (pICC422), for 8 h. Experiments were performed using tissue from four patients (aged between 41 and 181 months). Cryosections were incubated with rabbit anti-IRTKS (1:200) (Millard *et al.*, 2007), rabbit anti-Tir_EHEC_ (1:500) (Batchelor *et al.*, 2004; Girard *et al.*, 2007), rabbit anti-IRSp53 (1:50) (Weiss *et al.*, 2009) or mouse anti-cytokeratin (1:50, Dako) for 60 min at room temperature, washed and incubated in Alexa Fluor 488 or Alexa Fluor 647-conjugated secondary antibody (Molecular Probes) for 30 min. Counterstaining of bacteria and cell nuclei was performed using propidium iodide (Sigma). Sections were analysed with a Zeiss LSM 510 Meta confocal laser scanning microscope.

### Indirect immunofluorescence assay on mice colon sections

An indirect immunofluorescence assay using FFPE sections and cryosections fixed in 3% paraformaldehyde in PBS was used as previously described (Girard *et al.*, 2007; 2008). Appropriate sections were immunostained using the following antibodies: rabbit anti-O152 (kindly provided by Dr Lothar Beutin, The National Reference Laboratory for *E. coli*, Federal Institute for Risk Assessment, Berlin, Germany) was used to visualize *C. rodentium* on FFPE sections, chicken anti-intimin (kindly provided by Professor John M. Fairbrother, *E. coli* Laboratory, Faculté de médecine vétérinaire, Université de Montréal, Canada) was used to visualize *C. rodentium* in multilabelling procedures on cryosections (Girard *et al.*, 2005a), rabbit anti-Tir EHEC (Batchelor *et al.*, 2004; Girard *et al.*, 2007), rabbit anti-N-WASP (Girard *et al.*, 2009a), rabbit anti-Nck [Millipore (Upstate), Lake Placid, NY (USA)], rabbit anti-IRSp53 (Weiss *et al.*, 2009) and rabbit anti-IRTKS (Millard *et al.*, 2007) were used for multilabelling procedure on cryosections. Carbocyanine-2-conjugated donkey anti-chicken IgY, Phalloidin-Tetramethyl Rhodamine Iso-Thiocyanate-conjugated donkey anti-rabbit IgG and Rhodamine RedX-conjugated donkey anti-mouse IgG (Jackson ImmunoResearch Europe, Soham, Cambridgeshire, UK) secondary antibodies were used in respect of the primary antibodies. Phalloidin-Alexa Fluor 633 (Invitrogen, UK) was used to stain F-actin, while DNA of both bacteria and epithelial cells was counterstained with Hoechst 33342. Sections were examined with an Axio Imager M1 microscope (Carl Zeiss MicroImaging GmbH, Germany), images were acquired using an AxioCam MRm monochrome camera, and computer-processed using AxioVision (Carl Zeiss MicroImaging GmbH, Germany).

### Electron microscopy

Additional explants/tissue samples were processed for electron microscopy, as previously described (Girard *et al.*, 2007). Samples for scanning electron microscopy were examined without knowledge of the strain used, at an accelerating voltage of 25 kV using a JEOL JSM-5300 scanning electron microscope [JEOL (UK), Herts, UK]. Samples for transmission electron microscopy were observed using a Phillips 201 transmission electron microscope at an accelerating voltage of 60 kV (Philips, UK).

### Statistical analysis

Results are presented as a line plot (colonization) or a vertical bar chart (crypt length) with the mean and its standard deviation, or as a scatter plot with the median (CI). The non-parametric Mann–Whitney test and the non-parametric Kruskal–Wallis test with Bonferroni's corrected *a posteriori* comparisons were used to conduct pairwise and global statistical analysis, respectively, using commercially available GraphPad InStat v3.06 software (GraphPad Software, San Diego, CA, USA). A *P*= 0.05 was considered significant.
